# ATHLOS Healthy Ageing Scale score as the predictor of mortality in Poland and the Czech Republic: Magdalena Kozela

**DOI:** 10.1093/eurpub/ckac130.054

**Published:** 2022-10-25

**Authors:** M Kozela, A Pająk, JL Ayuso-Mateo, M Bobak, W Lu, H Pikhart, M Polak, A Sanchez-Niubo, U Stepaniak, JM Haro

**Affiliations:** 1 Department of Epidemiology and Population Studies, Jagiellonian University Medical College, Krakow, Poland; 2 Centro de Investigación Biomédica, CIBERSAM, Madrid, Spain; 3 Department of Psychiatry, Universidad Autónoma de Madrid, Madrid, Spain; 4 Department of Epidemiology and Public Health, University College London, London, UK; 5 Department of Social Psychology and Quantitative Psychology, University of Barcelona, Barcelona, Spain; 6 Recetox, Masaryk University, Brno, Czechia; 7 Research, Innovation and Teaching Unit, Parc Sanitari Sant Joan de Déu, Sant Boi de Llobregat, Spain

## Abstract

**Background:**

A novel tool to measure healthy ageing was developed by the ATHLOS consortium (Ageing Trajectories of Health-Longitudinal Opportunities and Synergies). ATHLOS Healthy Ageing Scale, constructed using harmonized data from 16 independent ageing cohorts, was designed to contribute to worldwide research on healthy ageing. The aim of the analysis was to assess the relation between ATHLOS Healthy Ageing Scale and all-cause mortality in Central European populations.

**Methods:**

Participants of the Polish and Czech HAPIEE cohorts (baseline age 45-69 years) were followed for 14 years. ATHLOS Healthy Ageing Scale was based on over 40 health indicators related to intrinsic capacity and functional ability. Cox proportional hazards models were used to determine the relationship between the ATHLOS Healthy Ageing Scale scores and all-cause mortality.

**Results:**

As many as 9,922 Polish and 8,518 Czech participants had non-missing data on the ATHLOS Healthy Ageing Scale score and mortality (1828 and 1700 deaths, respectively). After adjustment for age, dose-response associations with mortality in both genders and countries were found (HR for lowest vs. highest quintile of the ATHLOS Healthy Ageing Scale: 2.98 and 1.96 in Czech and Polish women and 2.83 and 2.66 in Czech and Polish men, respectively). Only modest attenuation was observed when additionally adjusted for education, economic activity, smoking and self-rated health.

**Conclusions:**

The ATHLOS Healthy Ageing Scale was found to be a good predictor of all-cause mortality in urban populations of Poland and Czechia. This composite indicator seems to be an important contributor to a better assessment of healthy ageing.

**Key messages:**

- Independent inverse associations between ATHLOS Healthy Ageing Scale and all-cause mortality were found in both men and women in urban population samples from Central and Eastern Europe.
- Given its predictive validity for all-cause mortality, this novel tool may substantially contribute to a better assessment of healthy ageing.

